# Supplementary material for: A Machine Learning-Driven Electrophysiological Platform for Real-Time Tumor-Neural Interaction Analysis and Modulation
Source: Nat Commun. 2026 Jan 7;17:49. doi: 10.1038/s41467-025-66988-y (PMC12780002; doi:10.1038/s41467-025-66988-y)
Supplement: Supplementary file 1 — Supplementary Information File [file 41467_2025_66988_MOESM1_ESM.pdf]

Supporting Information

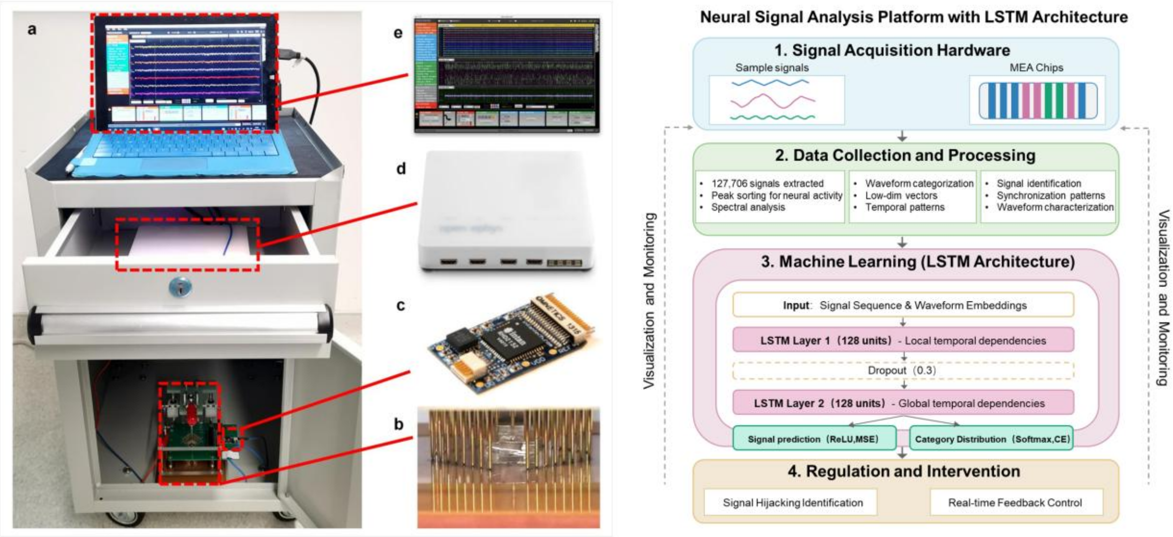

Figure S1. The entire platform design scheme.

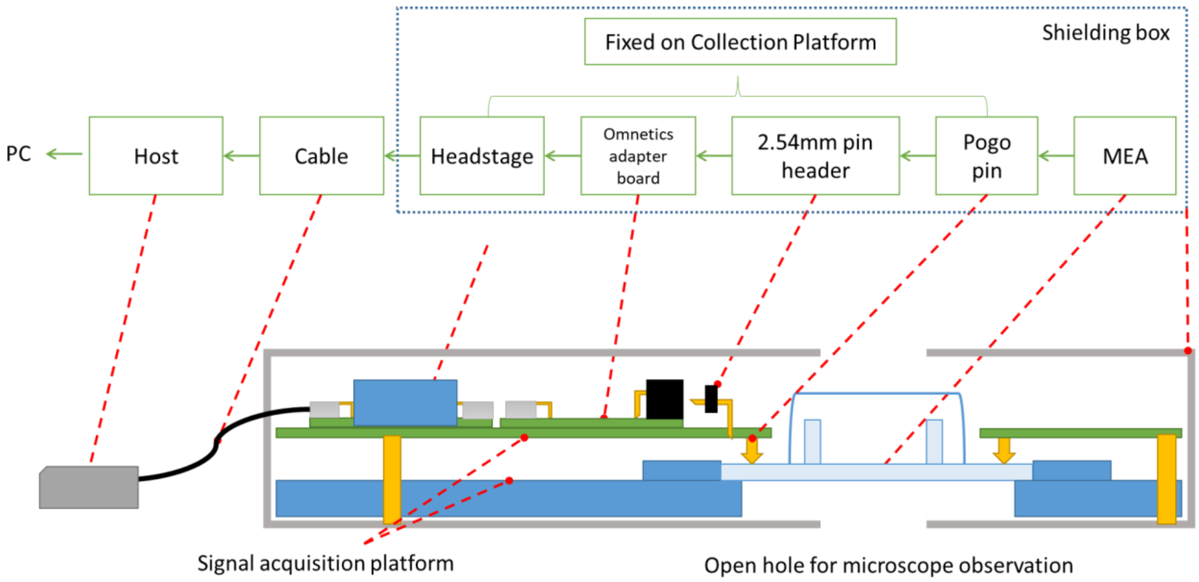

Figure S2. Design Schematic of the Customized Detection Platform.

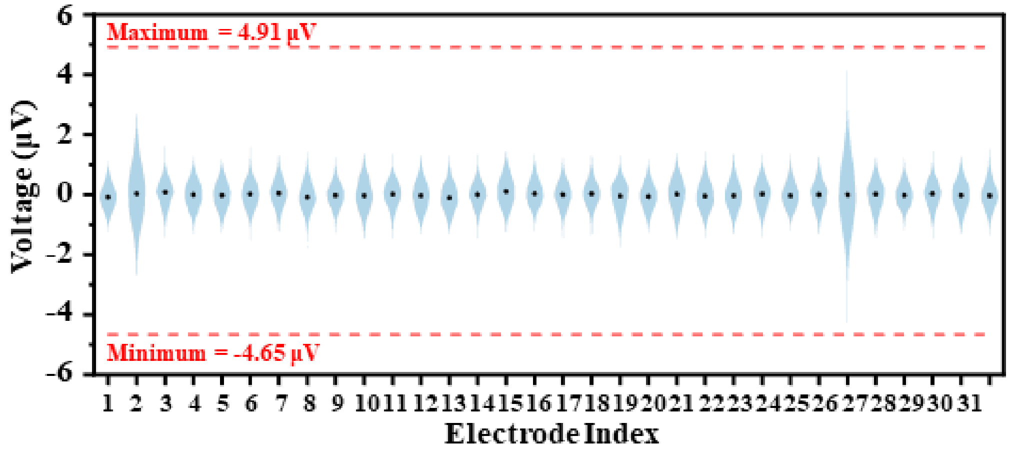

Figure S3. **Baseline noise voltage of a 32-channel electrophysiological monitoring system.**

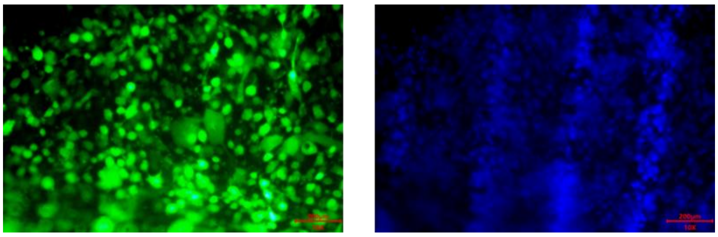

Figure S4. Cell viability after 7 days of culture.
Green: Active cells expressing GFP; Blue: DAPI staining. Scale bar: 200 μm.

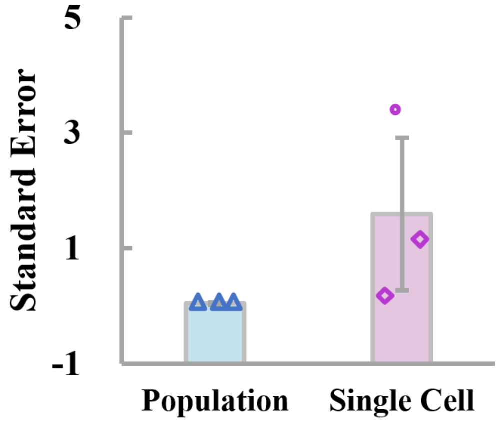

Figure S5. Comparison of Standard Error between population and single-cell tracking.

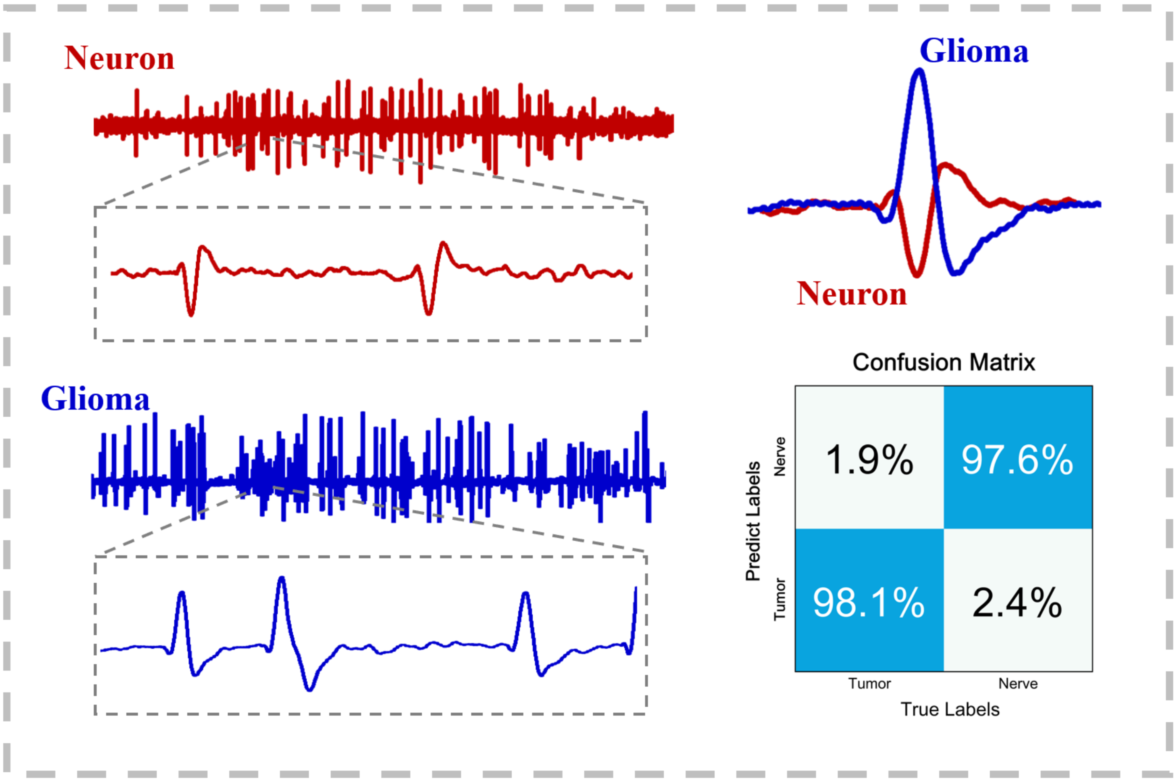

Figure S6. The distinct patterns of Neuron and Glioma signals along with a confusion matrix.

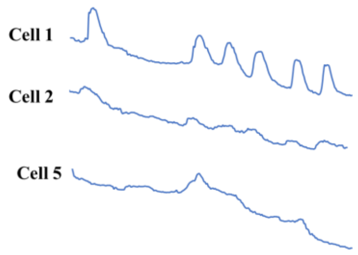

Figure S7. Signal display of Cells 1, 2, and 5.

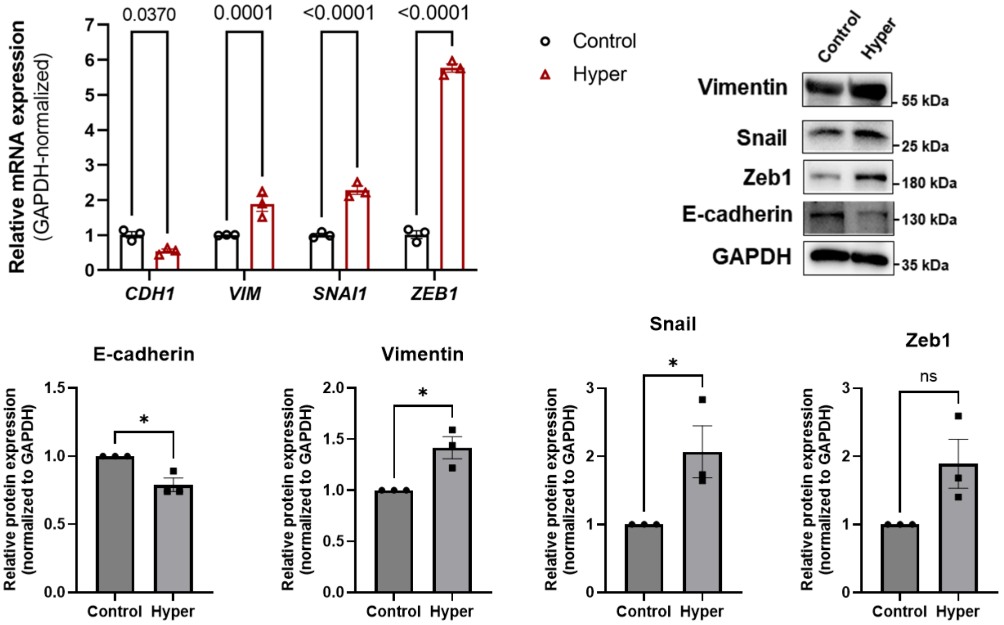

Figure S8. Invasion-related molecular signatures in hyper-invasive vs. control.

(n=3 biological samples, p-value determined by paired two-tailed paired t-test, *and n.s. indicate p-values < 0.01 and not significant, respectively, as determined by paired T-tests.).

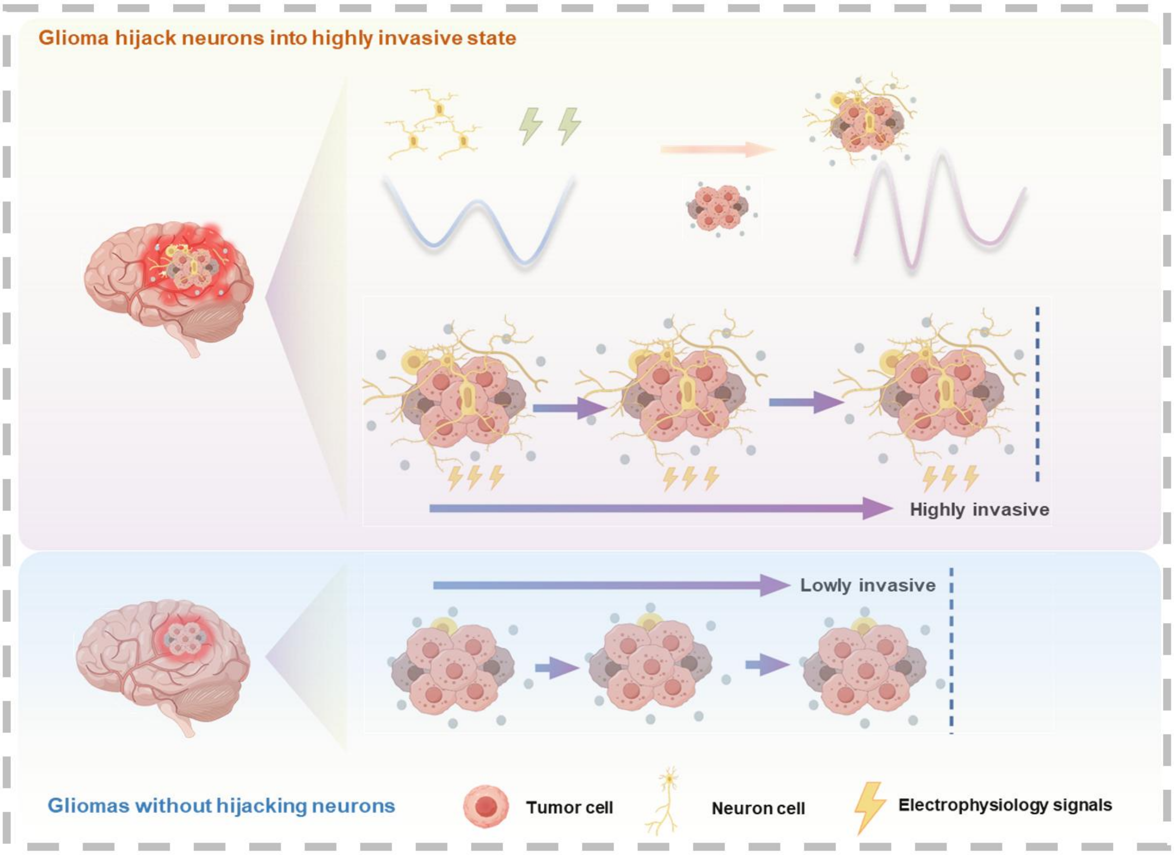

Figure S9. **Hypothesis of neuronal hijacking driving glioma hyper-invasiveness.**

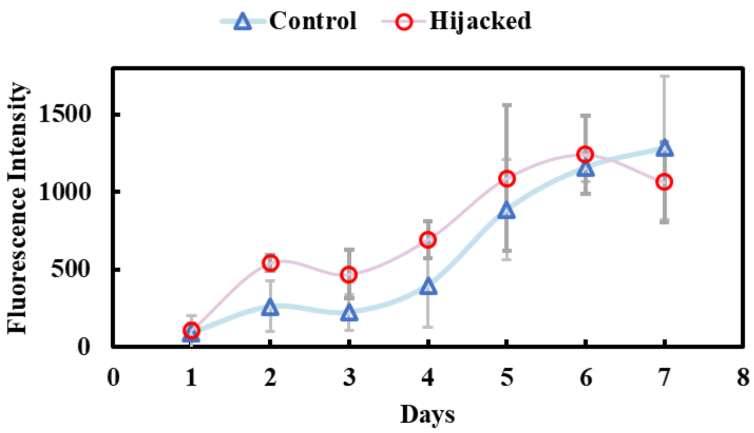

Figure S10. Cell proliferation comparison between control and hijacked cells.

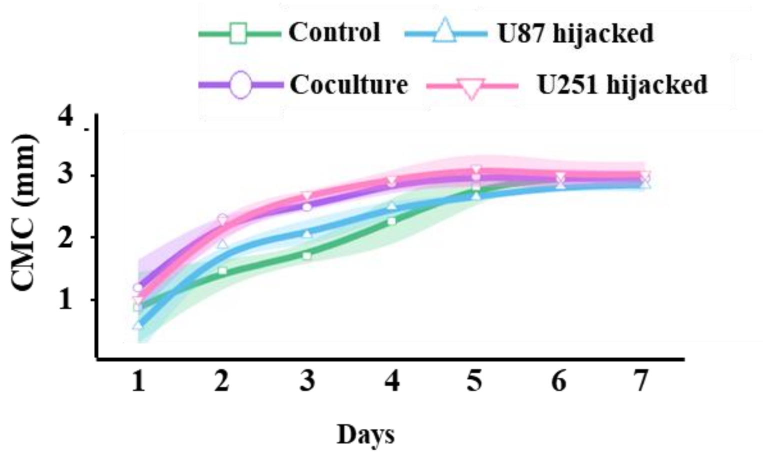

Figure S11. **Invasive behavior of human glioma U251 cells under four distinct bioelectrical conditions.** (Control, U87-hijacked signals, co-culture neural signals, or U251-hijacked signals).

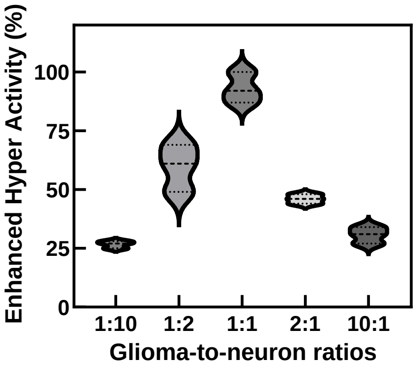

Figure S12. Glioma cell behavior across varying glioma-to-neuron ratios.

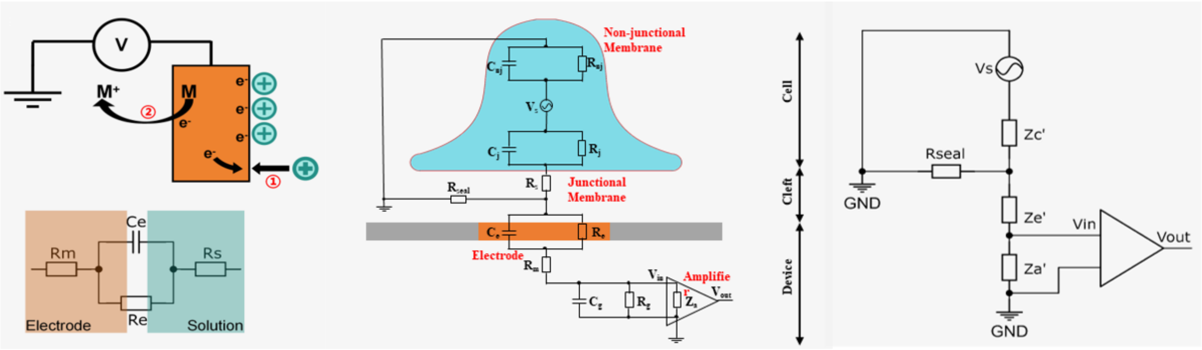

Figure S13. Cell-electrode coupling model for electrophysiology recording.

Table S1. The primers used in this study.

Pimers for RT-qPCR	Sequence (5’ to 3’)
CDH1-F	GAGTGCCAACTGGACCATTCAGTA
CDH1-R	CACAGTCACACACGCTGACCTCTA
VIM-F	AAGACGGTTGAAACTAGAGATGGAC
VIM-R	TGCTGGTAATATATTGCTGCACTGA
GAPDH-F	AGAAGGCTGGGGCTCATTTG
GAPDH-R	AGGGGCCATCCACAGTCTTC
SNAI1-F	GCTCCCTCTTCCTCTCCATACC
SNAI1-R	GGCAAGTTGATTGGAGGGATG
ZEB1-F	TTACACCTTTGCATACAGAACCC
ZEB1-R	TTTACGATTACACCCAGACTGC
