# Supplementary material for: A Machine Learning-Driven Electrophysiological Platform for Real-Time Tumor-Neural Interaction Analysis and Modulation
Source: Nat Commun. 2026 Jan 7;17:49. doi: 10.1038/s41467-025-66988-y (PMC12780002; doi:10.1038/s41467-025-66988-y)
Supplement: Supplementary file 2 — Description of Additional Supplementary Files [file 41467_2025_66988_MOESM2_ESM.pdf]

Description of Additional Supplementary Files

Supplementary Movie 1: Visualization of microfluidic control by the fluid control unit.
